# Natural Catalytic Antibodies in Norm, Autoimmune, Viral, and Bacterial Diseases

**DOI:** 10.1100/tsw.2010.98

**Published:** 2010-06-29

**Authors:** Georgy A. Nevinsky, Valentina N. Buneva

**Affiliations:** Institute of Chemical Biology and Fundamental Medicine of the Siberian Division of the Russian Academy of Sciences, Novosibirsk, Russia

**Keywords:** natural abzymes, healthy donors, autoimmune, viral, bacterial diseases

## Abstract

In human patients with autoimmune, viral, and bacterial diseases, the generation of antibodies (Abs) to foreign antigens and/or autoantibodies to self-antigens usually occurs. Some Abs with different catalytic activities (abzymes, Abzs) may be induced spontaneously by primary antigens and can have characteristics of the primary antigen, including the catalytic activity of idiotypic and/or anti-idiotypic Abs. Healthy humans usually do not develop Abzs or their activities are low, often on the borderline of sensitivity of the detection methods. Detection of Abzs was shown to be the earliest indicator of development of different autoimmune diseases (ADs). At the early stages of ADs, the repertoire of Abzs is usually relatively narrow, but it greatly expands with the progress of the disease, leading to the generation of catalytically diverse Abzs with different activities and functions. Some Abzs are cytotoxic and can play an important negative role in the pathogenesis of ADs, while positive roles have been proposed for other Abzs. Abzs with some low activities can temporarily be present in the blood of patients in the course of viral and bacterial diseases, but their activity increases significantly if these infections stimulate development of ADs. A significant increase in the relative Abz activities associated with a specific reorganization of the immune system, including changes in the differentiation and proliferation of bone marrow hematopoietic stem cells and lymphocyte proliferation in different organs. Different mechanisms of Abz production can be proposed for healthy externally immunized and for autoimmune mammals during the development of pathology.

### INTRODUCTION

Traditionally, antibodies (Abs) have been characterized as proteins produced by the immune system, which have the sole function of binding other molecules, called antigens (AG), with the goal of eliciting an immune response. In 1946, Linus Pauling noted similarities between the mechanisms of Ab-AG recognition and the interaction of transition states of catalytic reactions with enzymes and suggested a principal possibility of induction of catalytic Abs[1]. In 1986, two groups were able to produce the first artificial monoclonal Abs with catalytic properties, which were generated against hapten analogs of the transition states for *p*-nitrophenylphosphorylcholine[2] or for monoaryl phosphonate esters[3,4]. These catalytic Abs were termed abzymes (derived from antibody enzyme)(Abzs)[5,6,7,8,9]. Artificial Abzs against transition states of reactions catalyzing more than 100 distinct chemical reactions are novel biological catalysts that attracted much interest in the past years (for review, see [5,6,7,8,9,10,11,12,13]). Some artificial Abzs have been described that require cofactors for activity, similar to canonical enzymes[14]. The substrate specificity (and/or the specific activity) of some artificial Abzs is comparable to or even higher than that of enzymes with the same catalytic activity[15,16,17]. The mechanistic basis for the activity of such Abzs is presently well understood[13,18]. The field of artificial Abzs has been amply reviewed (see [5,6,7,8,9,10,11,12,13] and references therein).

The first example of a natural Abz was an IgG found in bronchial asthma patients that hydrolyzes vasoactive intestinal peptide (VIP)[19], the second was an IgG with DNase activity in systemic lupus erythematosus (SLE)[20], and the third was an IgG with RNase activity in SLE[21]. Sometimes viral and bacterial infections stimulate development of various ADs (see below). Later, a number of natural catalytic IgG, IgA, and IgM hydrolyzing DNA, RNA, nucleotides, and polysaccharides were detected in the sera of patients with several autoimmune, viral, and bacterial pathologies, and Abzs with these and other activities were discovered in the milk of healthy humans (for review, see [22,23,24,25,26,27,28,29]). Autoimmune, viral, and bacterial diseases were also found to stimulate production of IgG and/or IgM and IgA Abzs hydrolyzing different peptides and proteins, such as thyroglobulin (Hashimoto’s thyroiditis [HT] and rheumatoid arthritis)[30], prothrombin (multiple myeloma)[31], protein factor VIII (hemophilia A)[32], myelin basic protein (MBP)(multiple sclerosis [MS])[33,34,35], and viral reverse transcriptase and integrase (HIV-infected patients)[36,37]. Abzs with some other activities are described below.

Different methods of purification of electrophoretically homogeneous natural polyclonal and monoclonal Abzs (pAbzs and mAbzs, respectively) providing strong evidence that the enzymatic activity is an intrinsic property of Abs were developed and discussed in detail in several reviews[25,26,27,28,29].

In this review, new data concerning natural pAbzs and mAbzs with different catalytic activities in various autoimmune, viral, and bacterial diseases, their possible biological role in the pathogenesis of different pathologies, reasons for their catalytic heterogeneity, and possible uses of Abzs for diagnostics of ADs and as therapeutic agents are discussed.

### FEATURES OF THE IMMUNE STATUS OF PATIENTS WITH VARIOUS DISEASES

A special feature of ADs is high concentrations of auto-Abs to many different endogenous AGs[38,39]. The development of ADs is characterized by spontaneous generation of primary Abs to proteins, nucleic acids and their complexes, polysaccharides, nucleotides, etc.[40,41,42]. Later, the secondary idiotypic and then anti-idiotypic Abs to the primary ones are usually generated, and so on.

SLE is a systemic AD that is characterized by disorganization of conjunctive tissue with the paramount damage to skin and visceral capillaries[43]. Autoimmune and complex immunologic processes determined by genetic predisposition to immunoregulation disturbances are important in the pathogenesis of SLE[44]. The sera of SLE patients usually contain DNA and anti-DNA Abs in high concentrations[38,39]. Compared with healthy donors, concentrations of DNA and anti-DNA Abs are higher not only in patients with SLE (36% of SLE patients), but also in MS (17–18%), primary Sjogren's syndrome (18%), HT (23%), myasthenia gravis (6%), rheumatoid arthritis (7%)[45], autoimmune hepatitis[46], and also in lymphoproliferative[47] and some viral diseases (e.g., viral hepatitis and AIDS)[48]. In the sera of patients with several ADs, RNA and anti-RNA Abs were also detected[49,50,51,52].

Recently, 70 different clinical, biochemical, immunological, and blood cell characteristics used as indicators of SLE development and correlation between these indices were analyzed[53]. In comparison with healthy donors, an increased concentration of Abs was observed to various AGs (% of patients): DNA (60), cardiolipin (48), thyroglobulin (42), microsomal fraction of thyrocytes (MFT) (48), and rheumatoid factor (23)[53]. The titers of these auto-Abs were extremely variable in the group of 48 SLE patients and demonstrated relatively low correlation coefficient (CC) with many different characteristics. Therefore, this group was divided into subgroups with similar clinical manifestations using cluster analysis software; the resulting two subgroups contained 12 and 16 patients, while the remaining 20 individuals did not form groups with comparable characteristics. This led to an increase in some statistically significant CCs in the subgroup of 16 patients; for example, Abs to cardiolipin and to thyroglobulin (0.37 → 0.6); Abs to cardiolipin and to MFT (0.39 → 0.79), Abs to thyroglobulin and to MFT (0.77 → 0.93). Interestingly, some of the CCs in the subgroups of 12 and 16 patients were of the opposite sign, while there were no statistically significant correlations at all in the remaining 20 patients. Therefore, it is not surprising that there were no strictly consistent dependences or high CCs between different indices for the complete group of 48 patients. DNA- and RNA-hydrolyzing Abs was revealed in ~95% of SLE patients[25,26,27,28,29].

MS is a chronic demyelinating disease of the central nervous system. Its etiology remains unclear, and the most widely accepted theory of MS pathogenesis assigns the main role in the destruction of myelin to the inflammation related to autoimmune reactions[54]. Several recent findings imply an important role of B cells and auto-Abs against myelin auto-AGs in the pathogenesis of MS[54,55,56]. As in other ADs, anti-DNA Abs have also been detected in MS patients[45]. It was found that anti-DNA Ab is a major component of the intrathecal IgG response in MS patients and can promote important neuropathologic reactions in chronic disorders, such as MS and SLE[57].

The levels of anti-DNA and anti-MBP Abs in 18 and 95% of 49 MS patients, respectively, were significantly higher than in healthy donors[58,59,60], while Abzs with DNase and MBP-hydrolyzing activities were observed in 90–95% of the patients[25,26,27,28,29].

An analysis of correlation between the titers of Abs to DNA (and to MBP) and 13 different standard clinical parameters, including Poser criteria[61], was carried out[58,59,60]. For all 49 MS patients, the absolute CCs between Abs and 13 standard clinical parameters were relatively low and not statistically significant. Several CCs became remarkably higher after the cohort was divided into subgroups of patients with primary progressing, secondary progressing, and remitting course of the disease[58,59,60,61]. Interestingly, the CCs between the same two analyzed parameters can be either positive or negative in the case of different subgroups. The groups of remitting and secondary progressing course of MS patients were not "homogeneous" with respect to the patients' characteristics, and their further subdivision using cluster and factorial analysis revealed high, statistically significant, CCs[60].

Overall, the data indicate a complicated jigsaw of associations of the investigated parameters in different subgroups of SLE and MS patients[53,58,59,60]. This is in agreement with the diversity in the primary cause of pathology development, which can include exposure to a number of chemically active compounds or components of various viruses that can lead to damage of the immune system. In each SLE or MS patient, a transient error of the immune system may result from different genetic factors and/or specific environmental challenges. Therefore, it is not surprising that we could not find statistically significant correlation of titers of different Abs with the standard clinical characteristics in the whole groups of SLE and MS patients, since each patient can be characterized by an individual combination of genetic, environmental, chronic, inflammatory, autoimmune, demyelinating, neurodegenerative, and other factors[53,58,59,60].

The mechanism of autoimmune damage of the thyroid has not been adequately studied and the pathogenesis of the thyroid gland ADs remains unclear; different Abs are believed to play a significant role in the pathogenesis of HT[62,63]. There are several types of Abs in thyroid diseases: to thyroglobulin, to MFT, superficial AG, the second colloidal AG, thyreostimulating Abs, etc. Damage to thyrocytes by Abs has been suggested, and a pathogenic role of Abs to thyroglobulin and MFT has been proven[62,63,64]. A high concentration of anti-thyroglobulin Abs is one of the indicators of a systemic autoimmune process in patients with HT[65]. Anti-DNA Abs were revealed in 23% of HT patients[45].

In bronchial asthma, VIP, widely distributed in the central and peripheral nervous systems, acts as an auto-AG[19].

Microbial and viral infections expose the human organism to different components from the parasite's cells, including proteins, DNA, RNA, lipids, and polysaccharides. Some viral infections (AIDS, hepatitis, tick-borne encephalitis [TBE], etc.) can lead to the formation of Abs to various AGs, including DNA and RNA[66,67]. The blood of patients with viral infections often contains organ-unspecific auto-AGs. The sera of mammals infected with bacteria, parasites, and their pathogenicity factors contain a variety of Abs to the parasite's AGs and to human lipids, proteins, and nuclear components, including anti-DNA Abs[68,69,70,71,72,73]. The origin of anti-DNA Abs in the infections remains speculative; some of them may arise inadvertently in the course of a normal immune response due to the induction by Abs that bear structures (mimotopes) mimicking DNA[73]. It has been proposed that infectious agents can act in some cases through the mechanism of molecular mimicry and stimulate development of ADs. For instance, the agents responsible for molecular mimicry in MS may include measles, hepatitis B, herpes simplex, influenza, papilloma, and Epstein-Barr viruses[74].

It is known that different auto-Abs, including Abs to DNA and to different enzymes interacting with DNA, are present in the sera of not only autoimmune patients, but also healthy humans and other mammals[75,76]. Therefore, the fraction of patients with various ADs with the level of auto-Abs (including anti-DNA Abs) higher than in healthy humans usually does not exceed 6–35%[45,46,47,48]. Interestingly, healthy humans cannot develop Abzs with detectable DNase, RNase, and some other activities, while IgGs, IgMs, and IgAs hydrolyzing RNA, DNA, polysaccharides, peptides, and proteins have been revealed in autoimmune patients demonstrating even very low titers of auto-Abs comparable with those for healthy humans[25,26,27,28,29]. Since Abzs possess catalytic activity, in contrast to ELISA, it is possible to reveal even small amounts of Abzs with low activity in the pAbs pool due to the catalyst turnover and increase in the detection signal with time. Due to the higher sensitivity of Abzs detection, nuclease Abzs were revealed in 67% of HT and 90–95% of SLE and MS patients[25,26,27,28,29]. During pregnancy and immediately after delivery, women are very often characterized by immune processes similar to those in autoimmune patients ([25,26,27,28,29,77] and references therein). sIgA and IgG possessing DNase, RNase, amylase, ATPase, and several other unusual activities were found in the milk of 95–99% of lactating females[25,26,27,28,29].

It is well known that Abzs with different activities can be obtained after immunization of healthy animals. Therefore, it is not surprising that viral and bacterial infections can lead not only to generation of Abs to foreign AGs, but also to formation of catalytic Abzs. Interestingly, 31% of the TBE patients were shown to have an increased level of anti-DNA Abs, while detectable DNase activity of IgGs was revealed for 91% of these patients[78]. The relative activities of DNase Abzs in patients with different bacterial and viral infections was significantly lower than those for ADs and increased in the following order: streptococcal infection (erysipelas) < urogenital chlamydiosis associated with arthritis (Reiter's disease) < meningococcal meningitis < shigellosis < suppurative surgical infections caused by *Staphylococcus aureus* < suppurative surgical infections caused by epidermal staphylococci < urogenital ureaplasmosis associated with reactive arthritis ≤ TBE[78,79]. Abzs of different ADs and infected patients are different in their enzymic properties (see below). In contrast to autoimmune pathologies, nuclease Abzs are usually present in the sera of infected patients only temporarily and disappear soon after clearance of the infectious agent. Their activity can be significantly increased if viral or bacterial infections stimulate a development of autoimmune processes.

### THE ORIGIN OF ARTIFICIAL AND NATURAL ABZYMES

Artificial catalytic Abs can be obtained by immunization of animals with chemically stable analogs of transition states of chemical reactions (reviewed in [5,6,7,8,9,10,11,12,13]). On the other hand, artificial anti-idiotypic Abs can also possess catalytic activity[15,80]**.** If the active site of an enzyme plays the role of an AG triggering this anti-idiotypic chain, the secondary anti-idiotypic Ab can possess the structure, a part of which represents an "internal image" or "mold" of the active site of this enzyme, and, consequently, these Abs may possess some properties of this enzyme. This remarkable property of idiotypic mimicry has been exploited to raise anti-idiotypic mAbzs with acetylcholinesterase[81,82], carboxypeptidase[83,84], β-lactamase–like[85], and UreB-degrading activities[86].

The origin of natural Abzs in different ADs is complex. Similar to artificial Abzs against analogs of transition states of catalytic reactions, naturally occurring Abzs may be Abs raised directly against the enzyme substrates acting as haptens, which, bound to different proteins, could resemble transition states of catalytic reactions[25,26,27,28,29]. For example, autoimmune Abzs hydrolyzing different proteins are Abs against these proteins[19,23,24,25,26,27,28,29,30,31,32,33,34,35,87]. pIgGs from AIDS patients hydrolyzing HIV reverse transcriptase[36] and HIV integrase[37] were the first examples of proteolytic Abzs appearing in humans directly against these proteins due to a viral infection and immune reactions. However, in parallel with the formation of Abzs against viral proteins, the production of Abzs hydrolyzing human serum albumin and human casein specifically was revealed in HIV-infected patients[36].

To find out which AGs can induce Abzs with nuclease activities in ADs, we have immunized rabbits with DNA, RNA, DNase I, DNase II, and pancreatic RNase A[88,89,90,91,92]. In all cases, total Abs contained Abzs hydrolyzing both DNA and RNA, while Abs from nonimmunized rabbits did not hydrolyze nucleic acids[88,89,90,91,92]. Many SLE anti-DNA Abs are directed against DNA bound to proteins, including complexes with histones appearing as a result of internucleosomal cleavage during apoptosis[93]. Therefore, we have emulated such natural complexes using complexes of DNA and RNA with methylated bovine serum albumin (BSA). Immunization of rabbits with complexes of DNA and RNA with methylated BSA elicited production of 10- to 50-fold more active DNase and RNase IgGs[88,89]. In addition, it was shown that nuclease Abzs after the immunization of rabbits with DNase I, DNase II, and RNase contain both anti-idiotypic Abs against these enzymes and Abzs against nucleic acids complexed with these nucleases. Natural Abzs hydrolyzing DNA and RNA from the sera of patients with several autoimmune (SLE, MS, HT, polyarthritis, MS, etc.) and viral (viral hepatitis and AIDS) diseases demonstrated extreme diversity in their affinity for DNA and human nucleases[25,26,27,28,29]. Since immunization of autoimmune mice results in a dramatically higher incidence of Abzs with a higher activity than in conventionally used normal mouse strains[94,95], the formation of Abzs in ADs may be much more profuse.

Many autoimmune pathologies can be “activated” or “triggered” in clinically healthy women during pregnancy and soon after childbirth[96,97]. Independent of the presence or absence of detectable autoimmune reactions in women during pregnancy, postnatal autoimmune pathologies, such as SLE, HT, phospholipids syndrome, polymyositis, autoimmune myocarditis, etc., may emerge[77,96,97]. Autoimmunization of mothers during pregnancy similar to that occurring in autoimmune patients is very probable[25,26,27,28,29]. The relative activities of Abzs from human milk can be significantly greater than those of Abzs with the same activities from autoimmune patients[25,26,27,28,29]. Taking into account all these data, we believe that DNase and RNase pAbs of autoimmune patients and mother’s milk are “cocktails” of Abs against pure DNA and RNA and their complexes with various proteins, and anti-idiotypic Abzs to active centers of different DNA-hydrolyzing enzymes including complexes of these enzymes with DNA and RNA[25,26,27,28,29]. The central question is why autoimmunization of human mothers, autoimmune patients, and mice results in a dramatically higher incidence of catalytically inactive Abs and Abzs with enzyme properties as compared with healthy humans and animals.

### ASSOCIATION OF ABZYMES FORMATION WITH CHANGES IN COLONY FORMATION OF HEMATOPOIETIC PROGENITORS

MRL-lpr/lpr mice spontaneously developing a SLE-like disorder are a very promising model with which to study the mechanisms of generation of natural Abzs and their role in the pathogenesis of pronounced autoimmune disturbances. A mutation in the *lpr* gene of these mice leads to a deficit in functional *Fas* ligand and dysregulation of apoptosis in homozygotes[98,99]. As a result, the mice develop SLE-like phenotype, including accumulation of double-negative T cells ($CD4^{-}$ $CD8^{-}$ $B220^{+}$ $TCR^{+}$) in the peripheral lymphoid organs.

Recently, convincing evidence was provided that DNase, amylase, and ATPase activities are intrinsic to pIgGs from MRL-lpr/lpr mice, similar to Abzs from SLE patients[100,101,102,103]. The analysis of possible correlations between the relative catalytic activities of mouse IgGs in the hydrolysis of DNA, ATP, and oligosaccharides with several clinical and biochemical markers of autoimmune pathologies

(proteinuria, Ab titers to native and denatured DNA) at various stages of mouse SLE, pregnancy, and lactation was carried out[101,102,103]. It was shown that the appearance of pronounced visual symptoms (pink spots, cephalic and dorsal alopecia, general health deterioration, etc.) correlates well with proteinuria (≥3-mg/ml concentration of protein in urine)[101,102103]. The highest levels of anti-DNA Abs, DNase, ATPase, and amylase Abz activity, proteinuria, and visible markers of SLE were observed at 7–12 months of age, but we have used spontaneously diseased mice with all visible symptoms no older than 7 months. Although the state of "health" in the case of autoimmune-prone mice may be considered very provisional, the mouse SLE pathology is nevertheless spontaneous and autoimmune reactions leading to deep pathology develop gradually. In order to distinguish different levels of the pathology development, MRL-lpr/lpr mice demonstrating no typical SLE indices and Abz activities (similar to healthy control nonautoimmune mice) were conditionally designated (independently of age) as healthy MRL-lpr/lpr mice, whereas the animals demonstrating no visual or biochemical SLE indices, but having detectable Abz activities, were conditionally designated as prediseased mice[101,102,103]. The beginning of the lactation may be regarded as an important period associated with the production of Abzs[25,26,27,28,29]. Therefore, we have analyzed 12 groups of nonautoimmune mice and autoimmune-prone MRL-lpr/lpr mice with and without several pronounced SLE indices mentioned above, as well as pregnant and lactating mice, and assayed relative activities of their IgGs in the hydrolysis of DNA, ATP, and maltoheptaose (Tables 1 and 2).

It was shown that IgGs from the sera of 2- to 7-month-old control nonautoimmune CBA and BALB mice, and 2- to 3-month-old autoimmune-prone MRL-lpr/lpr mice (conditionally healthy mice), are catalytically inactive. During spontaneous development of deep SLE-like pathology, a specific reorganization of the immune system of these mice leads to conditions associated with a production of IgGs hydrolyzing DNA, ATP, and polysaccharides with low catalytic activities (conditionally prediseased mice). A significant increase in DNase, ATPase, and amylase IgG relative activities associated with the transition from prediseased to frankly diseased mice is correlated with additional changes in differentiation and proliferation of mouse bone marrow hematopoietic stem cells (HSCs) and lymphocyte proliferation in different organs (Table 2). In the prediseased mice, the higher number of CFU-GEMM (granulocytic-erythroid-megakaryocytic-macrophagic colony-forming unit) was accompanied by a striking increase in their size. Interestingly, after the mice spontaneously developed SLE associated with proteinuria and an increase in Abz relative activities, a significant increase in BFU-E (erythroid burst-forming unit) and a decrease in CFU-GM (granulocytic-macrophagic colony-forming unit) and CFU-GEMM colonies was observed. In contrast to the prediseased mice, the CFU-GEMM colonies of the diseased animals were of normal size. Thus, all mice demonstrating a detectable level of Abz activities without a significant increase in proteinuria and anti-DNA Abs can be considered prediseased; in comparison with conditionally healthy young mice, they are characterized by a specific profile of HSC differentiation, increased lymphocyte proliferation in the thymus and spleen, and increased apoptosis in the spleen (Tables 2–4).

The highest increase in all Abz activities was found in mice immunized with DNA, which, in comparison with prediseased and diseased mice, are characterized by a different profile of HSC differentiation and by suppressed apoptosis (Tables 1–4). The Abz production in spontaneously diseased mouse groups results from a specific balance of HSC proliferation and differentiation, lymphocyte proliferation, and apoptosis in different mouse organs[101,102,103]. It should be underscored that, in contrast to spontaneous mouse SLE after immunization of mice with DNA, the increase in Abz activities and development of an induced autoimmune pathology is not associated with remarkable changes in differentiation and proliferation of HSCs; only an increase in the rate of proliferation of lymphocytes in different organs and a significant decrease in apoptosis, especially in bone marrow, thymus, and spleen, where B-lymphocyte progenitors differentiate into Ab-producing plasmocytes[101,102,103], were observed (Tables 1–4).

**TABLE 1**
**Autoimmune Characteristics of Autoimmune-Prone MRL-lpr/lpr and Control Nonautoimmune Mice[101,102,103]**

| Group Description[†] | Group No. | No. of Mice | Urine Protein, mg/ml* | Abs to Native DNA, $A_{450}$* | Abs to Denatured DNA, $A_{450}$* | DNase Activity, %* | ATPase Activity, %[*] | Amylase Activity* |
|---|---|---|---|---|---|---|---|---|
| | | Control Males and Females | | | | | | |
| (CBA × C57BL) F1 (3–7 months) | 1 | 8 (4 females + 4 males) | 0.12 ± 0.07 | 0.04 ± 0.01 | 0.02 ± 0.01 | 0*** | 0*** | 1.0 ± 0.5*** |
| BALB/c (3–7 months) | 2 | 8 (4 females + 4 males) | 0.1 ± 0.08 | 0.03 ± 0.01 | 0.017 ± 0.004 | 0 | 0 | 1.1 ± 0.5 |
| | | MRL-lpr/lpr Males | | | | | | |
| Healthy (2–3 months) | 3 | 5 | 0.38 ± 0.1 | 0.032 ± 0.01 | 0.09 ± 0.07 | 0 | 0 | 1.9 ± 1.2 |
| Healthy, prediseased (7 months) | 5 | 5 | 0.8 ± 0.3 | 0.1 ± 0.05[+] | 0.16 ± 0.05 | **3.0 ± 1.0[‡]** | **0.4 ± 0.25** | n.d.[§] |
| Diseased (7 months) | 7 | 8 | **8.0 ± 3.1**** | **0.2 ± 0.05[+]** | **0.23 ± 0.11** | **22.0 ± 24.0** | **68.3 ± 98.0** | 3.7 ± 1.0 |
| Immunized | 9 | 6 | **9.5 ± 1.7**** | **0.6 ± 0.17** | **1.1 ± 0.16** | **360.0 ± 230.0** | **1333 ± 530** | **17.6 ± 7.5** |
| | | MRL-lpr/lpr Females | | | | | | |
| Healthy (2–3 months) | 4 | 5 | 0.31 ± 0.03 | 0.08 ± 0.03 | 0.12 ± 0.06 | 0 | 0 | 1.8 ± 1.1 |
| Healthy, prediseased (7 months) | 6 | 5 | **0.9 ± 0.2** | 0.18 ± 0.1[+] | 0.08 ± 0.04 | **6.1 ± 2.8** | **2.4 ± 1.7** | n.d. |
| Diseased (7 months) | 8 | 5 | **5.0 ± 3.8**** | **0.23 ± 0.1[+]** | 0.21 ± 0.12 | **20.0 ± 21.0** | **65.0 ± 93.0** | **9.2 ± 5.4** |
| Pregnant (2–3 months) | 10 | 5 | 0.31 ± 0.2 | **0.24 ± 0.05** | 0.25 ± 0.07 | **7.3 ± 6.0** | **39.3 ± 42.8** | **3.9 ± 3.6** |
| Lactating (3 months), 4 days after delivery | 11 | 5 | 0.32 ± 0.1 | **0.54 ± 0.3** | 0.35 ± 0.21 | **44.4 ± 40.6** | **367 ± 548** | **31.7 ± 27.3** |
| Lactating (3 months), 14 days after delivery | 12 | 5 | 0.70 ± 0.3 | **0.57 ± 0.28** | 0.39 ± 0.18 | **19.0 ± 24.0** | **191 ± 173** | **13.7 ± 11.0** |

* For each mouse, the mean and standard deviation of three experiments is shown.

** Proteinuria corresponds to ≥3 mg of total protein/ml of urine.

*** 100% relative activity corresponds to a complete transition of the substrate to its products of hydrolysis in the presence of 0.1 mg/ml IgGs .

§ Not determined.

† MRL-lpr/lpr mice demonstrating no typical SLE indices or Abz activities (similar to healthy control nonautoimmune mice) were conditionally designated healthy independently of age; the animals showing detectable levels of Abzs were conditionally designated as prediseased.

‡ Cohorts with statistically significant ($p \leq 0.05$) differences in the parameters in comparison with conditionally healthy MRL-lpr/lpr males and females are given in boldface.

\+ Values obtained using 10–12 mice.

Abz activities in the serum of pregnant females were comparable with those for prediseased mice, but the profile of HSC differentiation and cell apoptosis levels in pregnant and prediseased mice were different (Tables 1–4). Interestingly, pregnant females demonstrated the largest decrease in apoptosis in all organs and especially in the spleen. Thus, a significant decrease in apoptosis in the immunized and pregnant mice may be an important factor providing the increased number of specific lymphocytes producing auto-Abs and Abzs, which are normally eliminated[101,102,103]. Right after the beginning of lactation (4 days after delivery) and late in lactation (14 days after delivery), there was an observed increase in cell apoptosis and two different stages of significant change in the HSC differentiation profiles; the first stage was accompanied with a significant increase and the second, with a remarkable decrease in Abz activities (Tables 1–4). Overall, all mouse groups investigated are characterized by a specific relationship between Abz activities, HSC differentiation profiles, levels of lymphocyte proliferation, and cell apoptosis in different organs[101,102,103]. From our point of view, only the appearance of ATPase and DNase activities may be considered the earliest statistically significant marker of spontaneous mouse SLE, and a further significant increase in their activities correlates with the appearance

**TABLE 2**
**Formation of Bone Marrow Progenitor Colonies in Autoimmune-Prone MRL-lpr/lpr and Control Nonautoimmune Mice[101,102,103]**

| Group Description† | Visual Symptoms | Group No. | No. of Mice | No. of Colonies* | | |
|---|---|---|---|---|---|---|
| | | | | BFU-E | CFU-GM | CFU-GEMM |
| CBA (3–7 months) | No | 1 | 8 | 3.0 ± 0.5 | 7.3 ± 1.0 | 0.25 ± 0.05 |
| | | | | Males | | |
| Healthy (2–3 months) | No | 3 | 5 | 6.5 ± 1.5 | 7.0 ± 1.0 | 0.5 ± 0.1 |
| Healthy, prediseased (7 months) | No | 5 | 5 | **12.7 ± 1.4**‡ | **30.0 ± 1.3** | **9.2 ± 1.9** |
| Diseased (7 months) | Yes | 7 | 5 | **25.3 ± 9.8** | 7.4 ± 0.4 | **3.9 ± 2.0** |
| Immunized (3 months) | Yes, weak | 9 | 5 | 7.0 ± 2.1 | 6.0 ± 2.6 | 0.9 ± 0.7 |
| | | | | Females | | |
| Healthy (2–3 months) | No | 4 | 5 | 5.5 ± 0.5 | 11 ± 2.5 | 0.5 ± 0.2 |
| Healthy, prediseased (7 months) | No | 6 | 5 | **11.5 ± 2.0** | **23.0 ± 3.0** | **8.2 ± 3.0** |
| Diseased (7 months) | Yes | 8 | 5 | **22.1 ± 8.0** | 9.0 ± 3.9 | 2.4 ± 1.8 |
| Pregnant (3 months) | No | 10 | 5 | 6.8 ± 2.0 | **7.8 ± 1.5** | **0.1 ± 0.08** |
| Lactating (3 months), 4 days after delivery | No | 11 | 5 | 8.8 ± 2.0 | **19.1 ± 1.8** | 0.25 ± 0.2 |
| Lactating (3 months), 14 days after delivery | No | 12 | 5 | **21.0 ± 8.0** | 9.7 ± 0.5 | **2.1 ± 0.7** |

* For each mouse, the mean and confidence interval of four experiments is shown.

† MRL-lpr/lpr mice demonstrating no typical SLE indices or Abz activities (similar to healthy control nonautoimmune mice) were conditionally designated healthy independently of age; the animals showing detectable levels of Abzs were conditionally designated prediseased.

‡ Cohorts with statistically significant ($p \leq 0.05$) differences in the parameters in comparison with conditionally healthy MRL-lpr/lpr males and females are given in boldface.

of SLE visible markers and with an increase in the concentrations of anti-DNA Abs and urine protein. However, development of autoimmune reactions and the increase in the sera anti-DNA Abs and in the Abz activities in pregnant and lactating mice are not associated with visible SLE markers and proteinuria (Table 1).

The literature data and our findings suggest that ADs originate from specific changes in differentiation and proliferation of HSCs. From our point of view, the mechanisms of Abz production and the biological roles of Abzs in lactating females and in patients with autoimmune pathologies may be significantly different. During the last 2–4 months of pregnancy, the "immunomemory" in women "collects information" about all inside and outside compounds that can be harmful for infants, and produces Abs to these compounds after the beginning of lactation[26,27,28,29]. The changes in differentiation and proliferation of bone marrow HSCs and lymphocytes in other tissues and organs occurring in autoimmune patients cannot be easily normalized (these processes usually have a chronic character), while easy silencing of the "immunomemory" and a return of the specific immune processes to the norm in lactating women is a programmed phenomenon. In spite of the formal similarity of the immune system indices for the diseased and lactating mice, autoimmune reactions in lactating females are not associated with kidney dysfunction and proteinuria, and these groups can differ significantly in the specific cell composition of BFU-E, CFU-GEMM, and CFU-GM colonies and in clonal differentiation of the specific lymphocyte precursors in different mouse organs[101,102,103]. Nevertheless, our data also

**TABLE 3**
**Lymphocyte Proliferation in Mouse Organs[101,102,103]**

| Group Description† | Group No. | No. of Mice | Proliferation Level × $10^{-2}$, cpm* | | | | | | | |
|---|---|---|---|---|---|---|---|---|---|---|
| | | | Bone Marrow | | Lymph Nodes | | Thymus | | Spleen | |
| | | | – | +Con A | – | +Con A | – | +Con A | – | +Con A |
| CBA (3–7 months) | 1 | 8 | 28.1 ± 5.4 | n.d.** | 11 ± 3.2 | n.d. | 11 ± 2.8 | n.d | 40 ± 8.7 | n.d |
| Males | | | | | | | | | | |
| Healthy (2–3 months) | 3 | 5 | nd | nd | 12 ± 7.3 | 24.7 ± 16.3 | 5.6 ± 0.95 | 105 ± 22 | 13.7 ± 5.4 | 256 ± 59 |
| Healthy, prediseased (7 months) | 5 | 5 | nd | nd | 17.5 ± 3.4 | **157 ± 63‡** | 10.1 ± 3.7 | 179 ± 53 | **38.6 ± 2.2** | 405 ± 109 |
| Diseased (7 months) | 7 | 5 | 41 ± 15.6 | 66.6 ± 45.7 | 14.6 ± 9.1 | **249 ± 154** | 15.6 ± 9.3 | 160 ± 81 | 29.3 ± 10.6 | 300 ± 82.5 |
| Immunized (3 months o) | 9 | 5 | 37.6 ± 24.7 | 49.6 ± 35.7 | 20.4 ± 19.0 | **189 ± 144** | 7.0 ± 6.0 | 84 ± 61 | 33.0 ± 23.0 | 245 ± 163 |
| Females | | | | | | | | | | |
| Healthy, prediseased (2–3 months) | 4 | 5 | 25.4 ± 12.2 | 24.8 ± 10.8 | 4.4 ± 1.8 | 254 ± 169 | 2.7 ± 1.0 | 106 ± 33 | 7.1 ± 4.4 | 215 ± 83 |
| Diseased (7 months) | 8 | 5 | 23 ± 9.1 | 35 ± 10 | 7.7 ± 6.0 | 196 ± 58 | 7. 7± 4.5 | 186 ± 118 | 22 ± 11.8 | 246 ± 115 |
| Pregnant (2–3 months) | 10 | 5 | 36.8 ± 8.4 | 55.4 ± 23.7 | 10.3 ± 6.3 | 279 ± 85 | **18.2 ± 9.3** | 153 ± 57 | 5.1 ± 2.4 | nd |
| Lactating (3 months), 4 days after delivery | 11 | 5 | 44.9 ± 11.4 | **93.1 ± 29.4** | 6.6 ± 3.9 | 340 ± 162 | **8.8 ± 0.9** | 108 ± 35 | **44.4 ± 18.4** | **525 ± 115** |
| Lactating (3 months), 14 days after delivery | 12 | 5 | 15.8 ± 5.4 | 29.6 ± 10.6 | 7.1 ± 4.4 | 137 ± 57 | 6.7 ± 5.1 | 69 ± 66 | 27.1 ± 18.4 | 310 ± 159 |

* For each mouse, the mean and confidence interval of three experiments is shown.

** Not determined.

† MRL-lpr/lpr mice demonstrating no typical SLE indices or Abz activities (similar to healthy control nonautoimmune mice) were conditionally designated healthy independently of age; the animals showing detectable levels of Abzs were conditionally designated prediseased.

‡ Cohorts with statistically significant ($p \leq 0.05$) differences in the parameters in comparison with conditionally healthy MRL-lpr/lpr males and females are given in boldface.

support the hypothesis that the mechanisms of auto-Ab and Abz production in the state of disease and in pregnant or lactating females overlap to some extent[101,102,103]. The suppression of autoimmune processes in lactating females after the end of lactation implies the existence of special mechanisms, absent in autoimmune patients, which switch the immune system back to normal.

### BIOLOGICAL FUNCTION OF ABZYMES

Abzs have been studied primarily in the context of ADs where their biological role remains unknown: do they have a function or represent a dysfunction? It is quite possible that some Abzs play positive roles, while others are harmful. VIP-hydrolyzing Abs of asthma patients can have an important effect in the pathogenesis by decreasing the concentrations of VIP, which plays an important role in asthma pathophysiology[104]. Recently, it was shown that hMBP-hydrolyzing activity is an intrinsic property of IgGs, IgMs, and IgAs from the sera of MS patients[33,34,35], and the specific sites of the neural AG cleaved by Abs were established[105]. Recognition and degradation of MBP peptides by serum auto-Abs was confirmed as a novel biomarker for MS[106]. In MS, the protease activity of anti-hMBP Abzs can attack hMBP of the myelin-proteolipid sheath of axons. An established MS drug, Copaxone, appears to be a specific hMBP-hydrolyzing Abz inhibitor[105]. Consequently, the Abzs may play an important negative role in MS pathogenesis.

**TABLE 4**
**Apoptosis in MRL-lpr/lpr Mouse Organs in the Presence and in the Absence of Concanavalin A[101,102,103]**

| Group Description† | Group No. | No. of Mice | Apoptosis Level, Fluorescence (Relative Units)* | | | | | | | |
|---|---|---|---|---|---|---|---|---|---|---|
| | | | Bone Marrow | | Lymph nodes | | Thymus | | Spleen | |
| | | | – | +Con A | – | +Con A | – | +Con A | – | +Con A |
| CBA (3–7 months) | 1 | 8 | 19.8 ± 6.9 | nd** | 11.8 ± 6.5 | nd | 12.7 ± 6.4 | nd | 11.2 ± 7.5 | nd |
| Males | | | | | | | | | | |
| Healthy (2–3 months) | 3 | 5 | 19.0 ± 5.2 | nd | 11.5 ± 0.5 | 18.5 ± 1.0 | 12.2 ± 2.0 | 23.0 ± 3.0 | 10.0 ± 1.0 | 13.2 ± 3.0 |
| Healthy, prediseased (7 months) | 5 | 5 | 15.0 ± 4.2 | nd | 12.7 ± 2.0 | 18.5 ± 2.5 | 8.7 ± 1.2 | 24.3 ± 3.2 | 17.0 ± 2.1‡ | 21.3 ± 3.0 |
| Diseased (7 months) | 7 | 5 | 9.8 ± 2.3 | 14.6 ± 6.0 | 7.6 ± 2.6 | 9.1 ± 1.9 | 7.9 ± 4.2 | 14.6 ± 7.8 | 11.2 ± 1.9 | 15.6 ± 5.5 |
| Immunized (3 months) | 9 | 5 | 6.1 ± 2.3 | 8.7 ± 3.2 | 5.7 ± 2.6 | 9.8 ± 3.7 | 6.1 ± 4.2 | 11.2 ± 6.4 | 4.8 ± 1.9 | 10.8 ± 1.7 |
| Females | | | | | | | | | | |
| Healthy (2–3 months) | 4 | 5 | 17.8 ± 5.1 | 24.6 ± 6.1 | 15.2 ± 4.3 | 26.1 ± 8.7 | 14.4 ± 5.2 | 22.2 ± 7.3 | 19.5 ± 5.6 | 26.6 ± 8.4 |
| Diseased (7 months) | 8 | 5 | 15.8 ± 3.4 | 22.8 ± 5.2 | 12.2 ± 5.1 | 18.2 ± 4.5 | 11.2 ± 4.3 | 20.8 ± 4.9 | 13.2 ± 2.6 | 15.8 ± 4.6 |
| Pregnant (3 months) | 10 | 5 | 9.2 ± 2.7 | 16.2 ± 6.8 | 7.0 ± 2.3 | 9.5 ± 7.5 | 8.2 ± 1.9 | 13.2 ± 2.7 | 1.5 ± 0.5 | nd |
| Lactating (3 months), 4 days after delivery | 11 | 5 | 14.8 ± 4.4 | 17.3 ± 3.5 | 10.8 ± 4.5 | 9.2 ± 2.7 | 10.8 ± 4.6 | 14.0 ± 3.1 | 15.3 ± 2.8 | 20.0 ± 4.2 |
| Lactating (3 months), 14 days after delivery | 12 | 5 | 12.2 ± 6.5 | 13.3 ± 7.2 | 7.0 ± 2.5 | 9.2 ± 5.0 | 10.5 ± 4.0 | 11.7 ± 4.3 | 9.4 ± 4.3 | 11.8 ± 4.4 |

* For each mouse, the mean and confidence interval of three experiments is shown.

** Not determined.

† MRL-lpr/lpr mice demonstrating no typical SLE indices or Abz activities (similar to healthy control nonautoimmune mice) were conditionally designated healthy independently of age; the animals showing detectable levels of Abzs were conditionally designated prediseased.

‡ Cohorts with statistically significant ($p \leq 0.05$) differences in the parameters in comparison with conditionally healthy MRL-lpr/lpr males and females are given in boldface.

DNase Abzs from SLE, lymphoproliferative diseases[47], MS patients[28], and DNA-hydrolyzing Bence-Jones proteins from multiple myeloma patients[107] are cytotoxic, cause nuclear DNA fragmentation, and induce cell death by apoptosis. A decrease in Abzs hydrolyzing nucleic acids is most probably a positive sign in some diseases (SLE, MS, HT, etc.). For example, it was shown for HT patients that the relative activities of DNase Abzs correlate with the concentration of thyroid hormones and other biochemical and immunological indices of this pathology, and are related to the progressive deterioration of the clinical status, including exacerbation of thyroid gland damage[108]. The therapy of HT patients by thyroxine leads only to a temporary change in the hormone concentration in the blood, but did not affect the level of DNase Abs. However, treatment with the immunosuppressive drug Plaquenil (7-chloro-4[β-diethylamino-α-methylbutylamino]quinoline) significantly decreases the DNA-hydrolyzing activity of Abs, which correlates with rising thyroid hormone concentrations, enhanced thyroid gland function, and an improvement of the clinical state of the patients[108]. However, Abzs obtained by immunization of healthy animals, and from patients with various bacterial and viral infections, are not cytotoxic toward tumor and normal cells. Therefore, it is possible that the formation of cytotoxic nuclease Abzs is a consequence of specific immune processes in autoimmune patients associated with changes in the differentiation profile and the levels of proliferation of HSCs[101,102,103], and widening of the repertoire of Abzs in autoimmune mammals. Because of their ability to bind a variety of foreign AGs, natural pAbzs of patients with bacterial and viral infections can play a major role in the primary line of defense against infections. It was shown that the presence of IgGs with serine protease–like activity hydrolyzing small peptides in the serum strongly correlates with survival after sepsis[109]. In HT, Abzs hydrolyzing thyroglobulin have been considered a positive factor, since they could minimize autoimmune responses to thyroglobulin and prevent formation of immunocomplexes[110]. We have recently found that the serum of HIV-infected patients contains IgG fractions specifically hydrolyzing only viral integrase[37] or reverse transcriptase[36], but not many other tested proteins. Abzs hydrolyzing integrase significantly suppress 3'-processing and integration reactions catalysed by this enzyme. Abzs from AIDS patients also hydrolyze DNA[111]. The immune response to virus components is the most important factor slowing down the transition of HIV infection to the stage of AIDS[67]. Therefore, reverse transcriptase-, integrase-, and DNA-hydrolyzing Abzs may cooperatively protect HIV-infected patients from the development of AIDS.

Abs of healthy mammals possess superoxide dismutase[112,113], $H_2O_2$-dependent peroxidase, and $H_2O_2$-dependent oxidoreductase activities[114,115,116]. Therefore, it seems likely that some Abzs can reduce oxygen from $^{\bullet}O_2^-$ to $H_2O_2$, while other Abzs neutralize the latter mutagen. We suggest that the specific repertoire of mammalian pAbs with these activities can serve as an additional natural system of detoxification of reactive oxygen species, and can destroy toxic, carcinogenic, and mutagenic compounds[114,115,116].

Specific stimulation of production of various Abzs by the mother's immune system as a result of autoimmunization and infections may be a way of strengthening the protective function of breast milk Abs due to various Abz catalytic activities, and may play a very important role in the passive immunity of neonates and contribute to mucosal immunity by policing the function of some cells[25,26,27,28,29].

Taken together, it is obvious that the biological roles of various Abzs may be very different. In the early stages of ADs, the repertoire of Abzs is usually relatively small, but it greatly increases with the progress of the disease, leading to the generation of catalytically diverse Abzs with different activities and functions. In this respect, it should be mentioned that even pools of Abzs from autoimmune patients contain different sets of Abzs, which may be toxic or nontoxic toward different cells; the number of toxic sets increased with development of deep pathology. It may be a consequence of an extreme diversity of the variable fragments and active centers of various Abzs.

### EXTREME DIVERSITY OF ABZYMES

An extreme structural diversity of Abzs with various activities was revealed using a number of different methods[25,26,27,28,29]. It was shown that only the light chains of IgGs from the sera of asthma patients hydrolyze VIP[117]. The Abz-dependent hydrolysis of DNA and RNA by isolated light chains of Abs from human milk[118,119] and patients with SLE, MS, asthma, and other autoimmune patients, as well as from MRL-lpr/lpr mice, is more efficient than by intact Abs[28,29,100,120,121,122,123]. However, the catalytic center of recombinant variable fragment (scFv) of one DNase mIgG from MRL-lpr/lpr mice is located at the interface between the light and heavy chains, and both of these chains are able to hydrolyze DNA after separation[124]. The amylase activity of IgGs from MRL-lpr/lpr mice can be revealed in the intact IgG ($H_2L_2$) and its partially reduced forms ($H_2L$, $HL_2$, and HL), as well as in the separated H and L chains[101]. The separated L and H subunits of milk ATPase IgG are not capable of hydrolyzing ATP; this activity can be detected only for the intact IgG ($H_2L_2$) and for its partially reduced forms, $H_2L$ and HL[125], while isolated heavy and light chains of ATPase IgGs from MRL-lpr/lpr mice efficiently hydrolyze different nucleotides[102]. Thus, it is clear that light and heavy chains of different Abzs can contribute to the active sites of Abzs in different ways.

It was shown that IgGs, IgAs, and IgMs containing both $\lambda$ and $\kappa$ L-chains from milk of healthy mothers and patients with different ADs are active in the hydrolysis of different substrates[118,119,121, 122,123,126]. MS IgGs of all four subclasses, IgG1–IgG4, are catalytically active, with their contribution to the total MBP-hydrolyzing activity of Abzs increasing in the order: IgG1 (1.5–2.1%) < IgG2 (4.9–12.8%) < IgG3 (14.7–25.0%) < IgG4 (71–78%)[126], while contribution to DNase activity increases in the following order: IgG1 (6.9%) < IgG3 (9.3%) < IgG2 (18.2%) < IgG4 (65.6%)[127].

Canonical proteases belong to one of the following types: serine, thiol, acidic, and metal dependent. IgGs from the sera of patients with ADs, including VIP-hydrolyzing IgGs from asthma[19], casein-hydrolyzing Abzs from human milk[87], and from HIV-infected patients[36], are similar to serine proteases. It was shown that MS pIgGs and IgMs can contain Abzs with serine protease and metalloprotease activity[33,34,35], while Abs from HIV-infected patients specifically hydrolyzing viral integrase contain Abzs of four types resembling thiol, serine, acidic, and metal-dependent proteases, the ratio of which may be individual for every patient (Table 5)[37]. In addition, the immune system of HIV-infected patients can produce anti-integrase Abzs with a combined structure of the active center, which may carry amino acid residues typical of different proteases[37]. Altogether, it is obvious that catalytic Abs may be very different in their structural properties, which may change significantly depending on the Ab enzymic activity, specific autoimmune or viral disease, and even the patient analyzed.

**TABLE 5**
**Inhibition of Proteolytic Activity of Individual IgGs from 10 AIDS Patients by Specific Inhibitors of Proteases of Different Types[37]§**

| No. of Preparation | Inhibition, %* | | | | |
|---|---|---|---|---|---|
| | AEBSF | Leupeptin | Pepstatin A | Iodoacetamide | EDTA |
| 1 | 42 ± 5** | 74 ± 8 | 51 ± 5 | 85 ± 9 | 0 |
| 2 | 0 | 70 ± 7 | 0 | 83 ± 9 | 96 ± 9 |
| 3 | 0 | 0 | 0 | 66 ± 7 | 0 |
| 4 | 0 | 0 | 0 | 66 ± 5 | 44 ± 5 |
| 5 | 0 | 0 | 59 ± 6 | 98 ± 8 | 98 ± 8 |
| 6 | 0 | 0 | 0 | 87 ± 7 | 3 ± 1 |
| 7 | 0 | 11 ± 3 | 0 | 33 ± 4 | 45 ± 4 |
| 8 | 0 | 0 | 0 | 78 ± 8 | 40 ± 3 |
| 9 | 49 ± 5 | 0 | 0 | 12 ± 1.5 | 0 |
| 10 | 0 | 0 | 0 | 49 ± 5 | 2 ± 1 |

§ The inhibition of the proteolytic activity was estimated after incubation of the Abzs with specific inhibitors of serine- (AEBSF), acidic- (Pepstatin A), thiol-like (iodoacetamide), Me-dependent proteases (EDTA), and several different proteases (Leupeptin). However, since the proteolytic activity in several IgGs was summarily suppressed by specific inhibitors of serine, acidic, metal-dependent, and thiol proteases by more than 100%, it is possible that the immune system of HIV-infected patients produces anti-integrase Abzs with a combined structure of the active center, carrying amino acid residues typical of different proteases.

* The decrease in the intensity of the initial HIV integrase band estimated from SDS-PAGE electrophoresis data, the initial integrase intensity in the absence of inhibitor was taken for 100%.

** For each preparation, a mean and standard deviation of three experiments are shown.

Canonical enzymes usually specifically catalyze only one chemical reaction, whereas the same pIgGs, pIgAs, and pIgMs from the sera of patients with different autoimmune and viral diseases or human milk, as well as mouse monoclonal IgGs, possess both DNase and RNase activities and their relative activities in the hydrolysis of RNA is often 10- to 100-fold higher than in the hydrolysis of DNA[25,26,27,28,29, 119,121,122,128,129,130,131,132,133,134]. In contrast to known canonical ATPases, Abs from human milk and the sera of diseased MRL-lpr/lpr mice utilize not only ATP, but also other NTPs and dNTPs as substrates with comparable efficiencies[102,125].

It was interesting how monoclonal Abs possessing two or more different activities may be produced by individual immune systems. We have compared the affinity of pIgGs from the sera of nonimmunized rabbits and the animals immunized with DNA, RNA, DNase I, DNase II, and RNase A for DNA by chromatography on DNA-cellulose using the same conditions of chromatography[88,89,90,91,92]. Interestingly, the immunization by each of these five AGs induces a specific repertoire of Abzs. For example, anti-DNA and anti-RNA Abs did not contain Abz subfractions with a high affinity for DNA-cellulose (eluted with 3 M NaCl and acidic buffer), while the immunization of rabbits with DNase I, RNase, and DNase II results in the appearance of such subfractions; the $K_m$ and $k_{cat}$ values characterizing the hydrolysis of DNA and RNA were different for the eluted subfractions in the case of various AGs (e.g., Tables 6 and 7)[88,89,90,91,92].

**TABLE 6**
**Relative Content (RC) and Relative Total Activity (RA) of IgGs from Rabbits Immunized with Different Immunogens in the Hydrolysis of scDNA and poly(C) in Five Subfractions (Protein Peaks) Separated by Chromatography on DNA-Cellulose[88,89,90,91,92]***

| Peak No.[#] | Elution Conditions* | Nonimmunized | | DNA | | RNA | |
|---|---|---|---|---|---|---|---|
| | | RC, %** | RA, DNase (RNase), % | RC, % | RA, DNase (RNase), % | RC, % | RA, DNase (RNase), % |
| 0 | Nonfractionated | 100 | 0 (0) | 100 | 100 (100)* | 100 | 100 (100) |
| 1 | 0.0 M NaCl | 68.8 | 0 (0) | 22.5 | 87.4 (38.3) | 56.0 | 0 (0) |
| 2 | 0.1 M NaCl | 9.3 | 0 (0) | 5.7 | 12.6 (26.2) | 11.0 | 30.0 (0) |
| 3 | 0.5 M NaCl | 17.1 | 0 (0) | 66.6 | 0 (35.5) | 29.0 | 68.7 (100) |
| 4 | 3 M NaCl | 4.8 | 0 (0) | 5.2 | 0 (0) | 4.0 | 1.3 (0) |
| 5 | pH 2.6 | 0 | 0 (0) | 0 | 0 (0) | 0 | 0 (0) |
| | | **DNAse I** | | **DNase II** | | **Ribonuclease A** | |
| 0 | Nonfractionated | 100 | 100 (100) | 100 | 100 (100) | 100 | 100 (100) |
| 1 | 0.0 M NaCl | 37.3 | 9.3 (0) | 55.6 | 5.5 (19.7) | 45.0 | 6.0 (0) |
| 2 | 0.1 M NaCl | 18.2 | 29.5 (0) | 11.1 | 25.7 (52.9) | 22.6 | 67.5 (44.7) |
| 3 | 0.5 M NaCl | 34.2 | 54.6 (100) | 28.3 | 27.8 (27.4) | 15.8 | 6.4 (55.3) |
| 4 | 3 M NaCl | 0 | 0.0 (0) | 2.0 | 22.3 (0.0) | 7.8 | 11.3 (0) |
| 5 | pH 2.6 | 10.3 | 6.6 (0) | 3.0 | 18.6 (0.0) | 8.8 | 8.8 (0) |

* Electrophoretically homogeneous IgGs from healthy rabbits and animals immunized with DNA, RNA, DNase I, DNase II, and pancreatic RNase A were loaded onto a DNA-cellulose and proteins were eluted with Tris-HCl buffer, pH 7.5, containing different concentrations of NaCl (0.1–3 M) and then with 50 mM glycine-HCl, pH 2.6. Five IgG fractions were collected, dialyzed, and each fraction was used in the DNase and RNase activity assay.

** RC and RA are relative content of IgGs and relative activity of Abs in the case of every peak.

[#] Peak numbers are the same for all five chromatographies.

A very interesting question is why all these AGs can stimulate the formation of Abzs with RNase and DNase activities, while canonical enzymes usually possess only one of these activities. The immune system can theoretically produce up to $10^6$ different Ab variants in response to a single AG. However, chemical and structural patterns of DNA and RNA, as well as different nucleases hydrolyzing DNA and RNA, have a number of similarities. One can propose that $10^6$ possible Ab variants against one immunogen may vary in their closeness of fit to the structure of AG, some of them possessing better complementarity to other cognate AGs than to immunogen itself. In addition, spontaneous mutagenesis of lymphocyte Ig genes coding for Abzs could also produce Abs somewhat mismatched to the immunogen used.

**TABLE 7**
**$K_m$ and $k_{cat}$ Values Characterizing Five pIgG Fractions (Fig. 1) from the Sera of Rabbits Immunized with DNA, RNA, DNase I, DNase II, and RNase A in the Hydrolysis of Plasmid scDNA[88,89,90,91,92]***

| No. of the Fraction# | Conditions of IgG Elution from DNA-Cellulose** | Immunogen Used; Apparent $K_m$ and $k_{cat}$ Values in the Hydrolysis of Plasmid scDNA | | | |
|---|---|---|---|---|---|
| | | $K_m$, nM* | $k_{cat}$ x $10^4$, $min^{-1}$* | $K_m$, nM* | $k_{cat}$ x $10^4$, $min^{-1}$* |
| | | DNA | | RNA | |
| 0 | Nonfractionated | 58.9 ± 13.9 | 2.5 ± 0.3 | 20.0 ± 5.0 | 0.017 ± 0.002 |
| 1 | 0.0 M NaCl | 68.1 ± 11.4 | 6.9 ± 0.7 | 0 | 0 |
| 2 | 0.1 M NaCl | 21.5 ± 4.0 | 4.7 ± 0.4 | 21.0 ± 5.0 | 0.07 ± 0.02. |
| 3 | 0.5 M NaCl | 0.0 | 0.0 | 12. 0 ± 3.0 | 0.12.0 ± 0.2 |
| | | DNase I | | DNase II | |
| 0 | Nonfractionated | 8.8 ± 1.0 | 1.28 ± 0.02<br>0.28 ± 0.02 | 15.8 ± 2.0 | 1.0 ± 0.1 |
| 1 | 0.0 M NaCl | 68.7 ± 8.8 | 0.1 ± 0.015 | 60.8 ± 10.5 | 0.53 ± 0.08 |
| 2 | 0.1 M NaCl | 9.5 ± 1.5 | 0.28 ± 0.035 | 18.3 ± 3.5 | 2.2 ± 0.4 |
| 3 | 0.5 M NaCl | 7.0 ± 0.8 | 0.46 ± 0.07 | 13.5 ± 2.5 | 8.7 ± 1.7 |
| 4 | 3 M NaCl | 0.0 | 0.0 | 0.8 ± 0.1 | 3.9 ± 0.5 |
| 5 | pH 2.6 | 0.36 ± 0.05 | 0.16 ± 0.02 | 0.5 ± 0.09 | 1.1 ± 0.2 |
| | | RNase A | | | |
| 0 | Nonfractionated | 1.0 ± 0.4<br>15.4 ± 3.7 | 0.16 ± 0.05<br>0.61 ± 0.22 | | |
| 1 | 0.0 M NaCl | 18.3 ± 3.3 | 0.32 ± 0.04 | | |
| 2 | 0.1 M NaCl | 15.6 ± 1.6 | 4.7 ± 1.0 | | |
| 3 | 0.5 M NaCl | 11.3 ± 3.0 | 0.18 ± 0.03 | | |
| 4 | 3 M NaCl | 0.8 ± 0.1 | 0.3 ± 0.05 | | |
| 5 | pH 2.6 | 0.5 ± 0.1 | 0.45 ± 0.06 | | |

* Five fractions for every immunogen were obtained by affinity chromatography of total pIgGs on DNA-cellulose (see comments to Table 6).

** Salt concentrations of the buffer used for elution of the IgG fraction from DNA-cellulose are shown.

## Peak numbers are the same for all five chromatographies.

The affinity of scDNA for DNase IgGs from rabbits immunized with DNA, RNA, DNase I, DNase II, and RNase in terms of $K_m$ (0.5–60.8 nM, Table 7) is comparable with that for IgGs from SLE ($K_m$ = 43–92 nM)[135] and MS patients (0.36 nM)[123], and is three to four orders of magnitude higher than that of DNase I ($K_m$ = 46–58 μM)[135]. The data concerning Abzs from the sera of patients with different ADs were indicative of catalytic and affinity heterogeneity of nonfractionated pAbs. We have analyzed the possible diversity of Abzs in their affinity for different substrates by chromatography on sorbents bearing the immobilized substrates. Fig. 1 shows the profile of chromatography of pIgGs of a diseased MRL-lpr/lpr mouse on DNA-cellulose[136]. A similar distribution of human milk Abzs with protein kinase (Fig. 2)[137,138], lipid kinase[139,140], polysaccharide kinase[141,142], and nucleotide-hydrolyzing[125] activities upon their affinity chromatography on ATP-Sepharose was observed. When IgGs from SLE and MS patients[121,122,123], diseased MRL-lpr/lpr mice[136], milk of healthy human mothers[119,128], and rabbits immunized with DNA, RNA, DNase I, DNase II, and RNase A[88,89,90,91,92] are eluted from

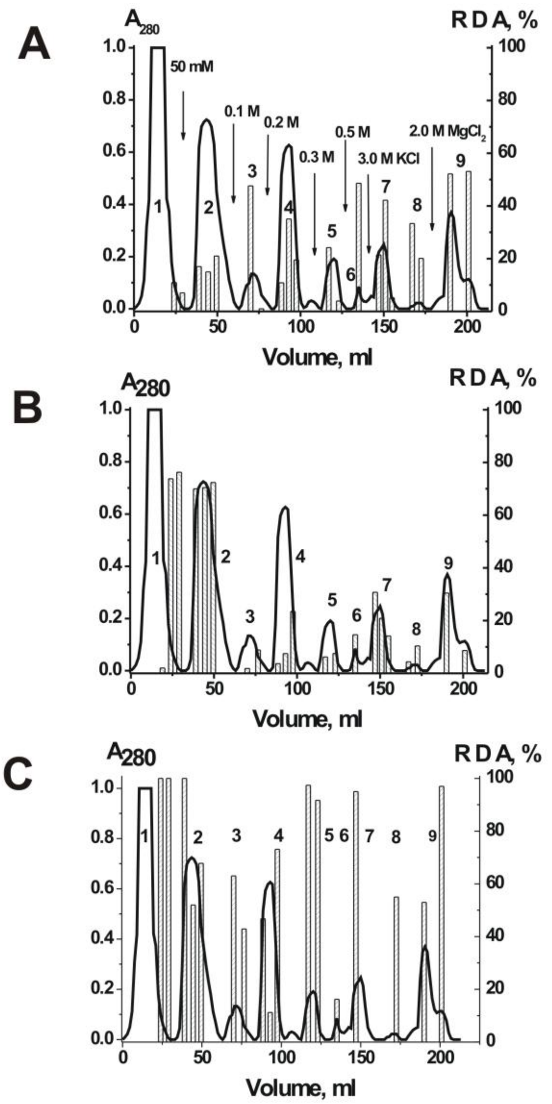

**FIGURE 1.** Affinity chromatography of a pIgG preparation from the serum of a diseased mouse with high proteinuria on DNA-cellulose: (—), absorbance at 280 nm (A,B,C)[136]. The relative DNase activity (RDA) of the IgG fractions eluted from the resin by different concentrations of KCl and 2 M $MgCl_2$ in the presence of $Ca^{2+}$ (A), $Mg^{2+}$ (B), and $Mn^{2+}$ ions (C) is shown by the bars. The relative activity was normalized to standard conditions corresponding to 5 µl of the eluate and 10 min of incubation; the highest RDA among the fractions determined in the presence of $Mn^{2+}$ ions (C) was taken for 100%. Errors in the initial rate determination from three experiments for any $Me^{2+}$ did not exceed 7–12%.

DNA-cellulose by a KCl gradient (0–3 M), the protein, DNase, and RNase activities are distributed all over the chromatography profiles. In all cases, we found specific Ab subfractions with very high affinity for DNA, which are eluted from DNA-cellulose only with 2–3 M $MgCl_2$ or with an acidic buffer (pH 2.6). Abzs with casein-[36,87], MBP-[33], reverse transcriptase-, and integrase-hydrolyzing[87] activities also demonstrate distribution of Abzs all over the profiles under chromatography on the sorbents with the immobilized cognate proteins. The affinity of Abz fractions for all immobilized substrates increases

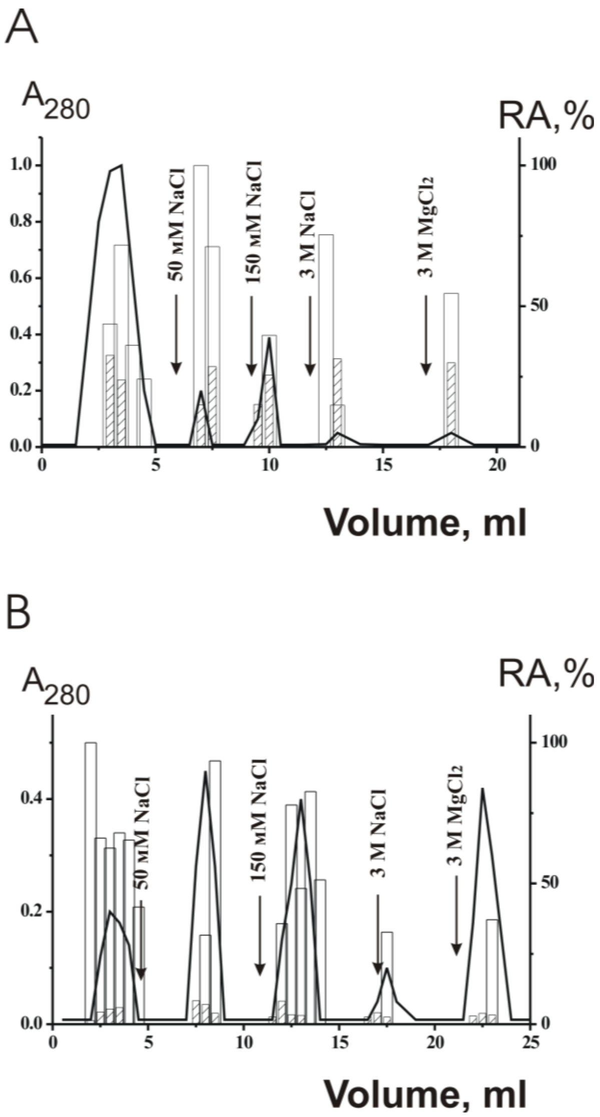

**FIGURE 2.** Affinity chromatography of polyclonal human milk IgGs (A) and sIgAs (B) on ATP-Sepharose: (—), absorbance at 280 nm (A and B)[139]. The relative lipid kinase activity (RA) of the Ab fractions eluted from the resin by different concentrations of NaCl and 3 M $MgCl_2$ was estimated in the presence of [$\gamma$-$^{32}$P]ATP and [$^{32}$P]orthophosphate (white and gray bars, respectively). The highest RA among the fractions was taken for 100%.

gradually with the increase in the eluting NaCl concentration (e.g., Tables 6 and 7). More often, at the onset of ADs, one can reveal only one or two pairs of $K_m$ and $V_{max}$ values for the substrate analyzed, reflecting the presence of only one or two monoclonal fractions of Abzs in the IgG preparations[25,26,27,28,29]. Then, after a prolonged period of pathology, pAbs usually demonstrate several well-distinguishable $K_m$ and $V_{max}$ values. During an exacerbation of ADs or after a very long period of chronic AD conditions, the number of monoclones producing different Abzs and, as a consequence, the number of $K_m$ and $V_{max}$ values may be very large; usually, it is impossible to establish how many values of these parameters in total characterize the full repertoire of Abzs making up the pAbs pool of these patients (e.g., Fig. 3). All these data are indicative of the extreme diversity of Abzs with different activities in their affinity for specific substrates.

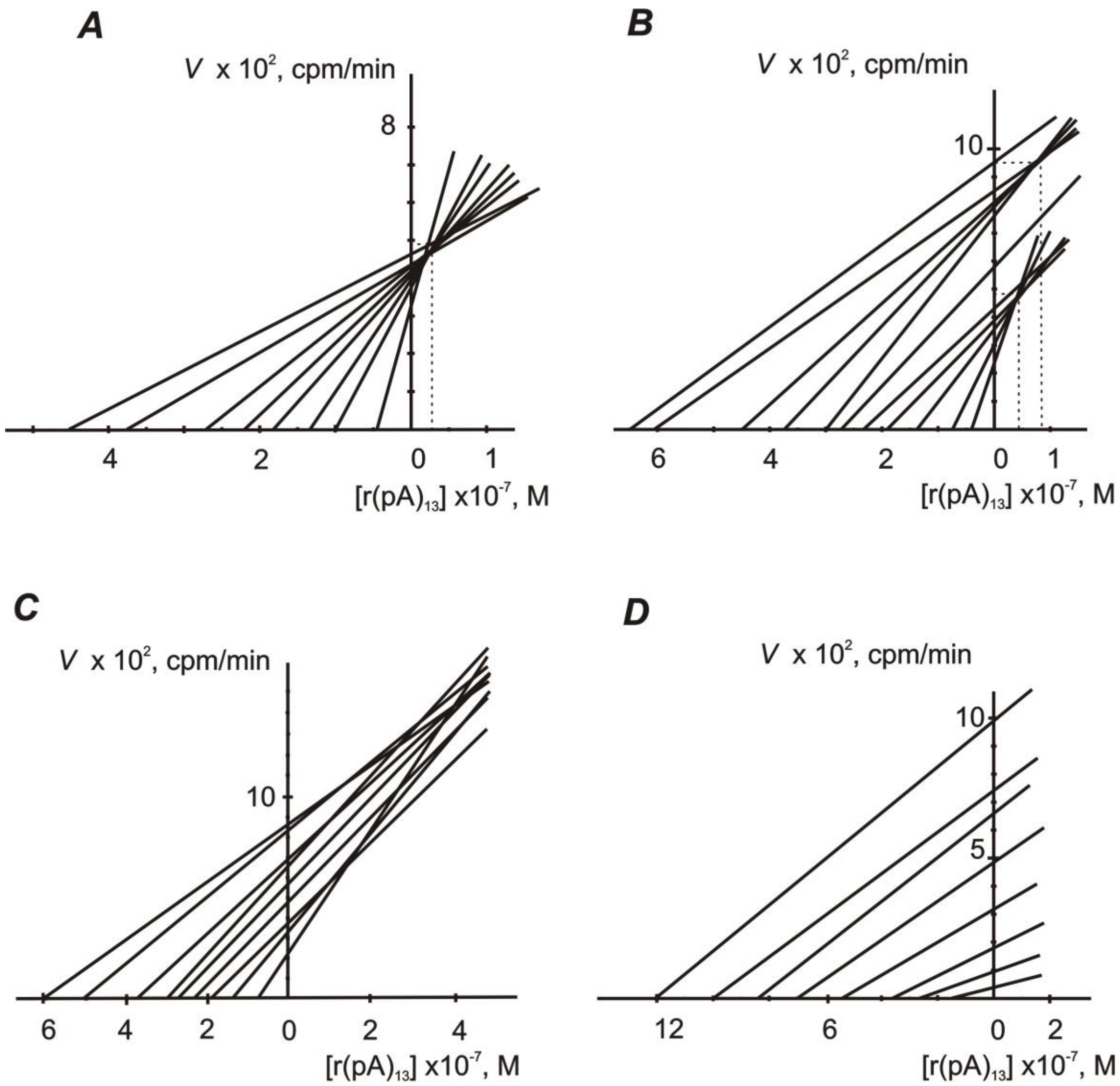

**FIGURE 3.** Initial rates of IgM-dependent hydrolysis of $[5'\text{-}^{32}P](pA)_{13}$ as a function of its concentration for Ab preparations from sera of four different SLE patients[121]. The $K_m$ and $V_{max}$ values were determined from Cornish-Bowden plots (A–D). The common intersection point gives the values of $K_m$ and $V_{max}$ in the case of IgM-1 (A) and two values of both $K_m$ and $V_{max}$ values for IgM-2 (B); a set of intersection points for $(pA)_{13}$ shows a range of $K_m$ and $V_{max}$ values of IgM-3 (C); no obvious intersection points are evident in the IgM-4 preparation (D).

The catalysis mediated by artificial Abzs is usually characterized by relatively low reaction rates: $k_{cat}$ values are $10^2$- to $10^6$-fold lower than for canonical enzymes[13]. The known $k_{cat}$ values for natural Abzs from autoimmune patients catalyzing many different chemical reactions vary in the range of 0.001–370 $min^{-1}$ ([25,26,27,28,29] and references therein). As we have shown, the fraction of pAbzs with different catalytic activities in the serum of autoimmune patients usually does not exceed 1–7% of total Igs[25,26,27,28,29]. The measured apparent $k_{cat}$ values usually characterize the relative content of catalytically active Abzs subfractions in the total pool of physically homogeneous pAbs. Since pAbzs usually contain a major subfraction (or subfractions) characteristic of each pathology and the individual patient, the relative activities of nonfractionated Abs typically characterize the activity of the major subfraction with the highest activity[25,26,27,28,29]. DNase pAbzs from ADs and from human milk usually demonstrate relatively low specific activities, 0.001–5% of those of known human DNases, Abzs from human milk usually being more active[29]. However, the relative activities of Abs from the sera of autoimmune patients vary markedly from patient to patient; in many patients, the relative activities for DNase Abs are comparable with those for restriction nucleases and repair enzymes[29,135].

Fig. 4 illustrates a range of possible relative DNase activities (2 h of incubation) in patients with different autoimmune and viral diseases. The percentage of patients with low (only formation of relaxed DNA), intermediate (partial DNA degradation), and high DNase activity (full DNA degradation) usually significantly changes from one pathology to another ([25,26,27,28,29] and references therein). Interestingly, the specific DNase activity of IgMs from the sera of SLE and MS patients usually exceed those of IgG five- to 20-fold[121,122]; a similar situation was observed for MS IgMs and IgAs with MBP-hydrolyzing activity[34]. Overall, the relative DNase activity of Abs is strongly dependent on the individual patient or immunized animal, but generally increases in the following order: immunized healthy rabbits ≤ diabetes < bacterial infections < TBE < autoimmune hepatitis < polyarthritis <AIDS ≤ HT < MS ≤ SLE.

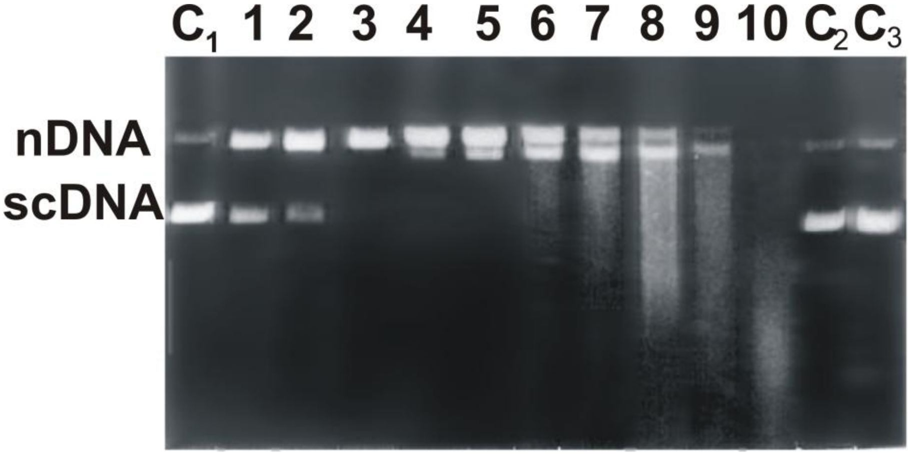

**FIGURE 4.** DNase activities of catalytic IgGs from patients with different ADs in the cleavage of supercoiled (sc) and nicked (n) plasmid DNA. Lanes 1–10, IgGs from the sera of 10 different patients; $C_1$, scDNA incubated alone; $C_2$ and $C_3$, scDNA incubated with Ab from the sera of two healthy donors[25,26,27,28,29,108,120,122,123,130,144].

The specific activities of RNase IgGs and IgMs from the sera of patients with different ADs may be 10- to 200-fold higher than those of DNase Abzs from the same patients. RNase activities of autoimmune Abzs are 0.1–20% of that of RNase A and of six known human sera RNases, while poly(A) is hydrolyzed by autoimmune Abzs two to 10 times faster than by RNase A, one of the most active RNases known[21,121,122,130,131,143,144]. The specific activity of Abs of several MS patients was 40–400% of that of RNase A[144]. Among the most active are Abzs from MRL-lpr/lpr mice hydrolyzing different nucleotides; their $k_{cat}$ values vary in the range 82–370 $min^{-1}$[102]. The $k_{cat}$ values characterizing Abzs with various enzymatic activities that have different affinity for immobilized substrates as estimated by affinity chromatography can differ up to $10^4$-fold. Thus, Abzs with different activities and from various sources demonstrate an extreme diversity in their $k_{cat}$ values and affinities for the cognate substrates.

Many canonical proteases can hydrolyze all proteins, while Abzs from the sera of patients with autoimmune and viral diseases usually specifically hydrolyze only the antigen – VIP (asthma and healthy donors[19]), thyroglobulin (HT, rheumatoid arthritis, and healthy donors[30]), casein (milk of healthy mothers and HIV-infected patients[36,87]), MBP (MS[33,34,35]), HIV reverse transcriptase and integrase (Fig. 5; AIDS)[36,37]), and human serum albumin and casein (HIV-infected patients[36]) – but not other tested proteins. The patterns of hydrolysis of the same protein by Abzs specific to this protein and by canonical proteases are usually quite different[36,87]. However, there may be several epitope peptide sequences, which can be hydrolyzed by pAbzs. pIgGs from the sera of two asthma patients hydrolyzed six

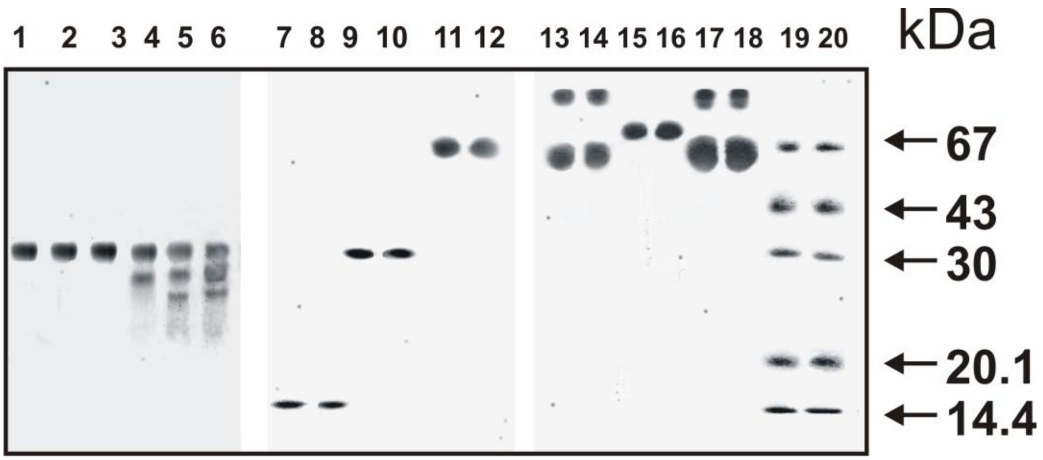

**FIGURE 5.** SDS-PAGE analysis of protein hydrolysis by AIDS pIgGs[37]. HIV-1 integrase (0.2 mg/ml) was incubated for 16 h in the absence (lane 1) or in the presence of 0.1 mg/ml pIgGs from two healthy donors (lanes 2 and 3) and pIgGs from two AIDS patients (lanes 4 and 5), or with 0.01 mg/ml AIDS IgGs purified on Integrase-Sepharose (6). Preparations of other proteins (0.2–0.4 mg/ml) were incubated for 16 h without (odd numbers) or with 0.05 mg/ml AIDS IgGs purified on Integrase-Sepharose (even numbers): lysozyme (7 and 8); human milk casein (9 and 10), p66 HIV-1 reverse transcriptase (11 and 12); human serum albumin (13 and 14); human milk lactoferrin (15 and 16); BSA (17 and 18); a mixture of protein molecular mass markers (19 and 20).

peptide bonds localized between amino acid residues 14 and 22 in VIP, while IgGs from the second patient cleaved an additional peptide bond between the residues 7 and 8[145]. AIDS IgMs hydrolyzed virus envelope gp120 protein within several peptides[146]. Four sites of MBP cleavage are localized within four different immunodominant MBP regions[105].

Fractions of Abzs from human milk[147] and from the sera of patients with different ADs[147,148,149,150,151] demonstrate different modes of action in the hydrolysis of malto-oligosaccharides, *p*-nitrophenyl malto-oligosaccharides, and *p*-nitrophenyl α-D-glucopyranoside; several samples of Abzs demonstrated β-xylosidase activity, which is not observed in known mammalian polysaccharide-hydrolyzing enzymes.

Interestingly, pAbzs from different ADs usually hydrolyze both single- and double-stranded DNA with comparable efficiency, but the hydrolysis of double-stranded plasmid DNA is often much faster[25,26,27]. The oligonucleotide (ODN) cleavage patterns are distinct for different MS patients[123]: some Abzs are sequence dependent, whereas others produce both 5'-phosphate-terminated products like those of DNase I and 3'-phosphate-terminated products typical for DNase II, or hydrolyze hetero-ODN in a sequence-independent manner. pIgGs from various MS patients demonstrate different combinations of endo- and exonuclease activities, but the properties of the DNase Abzs distinguish them from other known DNases[123].

We have shown that the specificity of pIgGs from patients with HT, polyarthritis[130], SLE[21], MS[144], and different types of hepatitis[143] for typical homopolynucleotide poly(N) substrates, cCMP, and $tRNA^{Phe}$ with a compact stable structure[130,131,132,133] is different, correlates with the disease, and is distinguishable from the specificity of known human RNases. Table 8 demonstrates the difference in average RNase relative activities of IgGs from the sera of patients with different autoimmune and viral diseases in the case of different polynucleotide substrates. A similar situation is observed for IgMs from patients with various ADs[130,131,132,133]. The RNase activity of IgGs from patients with different ADs is specifically stimulated by $Mg^{2+}$ ions, which nearly completely suppress the activity of all known human RNases[130,131,132,133]. In contrast to the typical substrates, no correlation between the IgG cleavage specificity and a specific disease can be revealed in the case of tRNA with a flexible structure; each patient demonstrates an individual repertoire of RNase pIgGs independently of the disease and an individual pattern of $tRNA^{Lys}$ hydrolysis[131,132]. Under certain conditions, some RNase Abzs exhibit

**TABLE 8**
**Relative Specific Activities of pIgGs from the Sera of Patients with Different ADs in Comparison with those for Pancreatic RNase A and RNases from Human Blood in the Hydrolysis of Various Ribopolynucleotides and cycloCMP[29,122,29,130,143]**

| **Preparation** | **Relative Specific Activities, %*** | | | | |
|---|---|---|---|---|---|
| | **cCMP** | **poly(U)** | **poly(A)** | **poly(C)** | **Total RNA** |
| | | | RNases | | |
| RNase A | 100 | 2 *(200)*** | 0.01 | 100 *(10000)* | 15 *(1500)* |
| Human RNases[§] | nd*** | 3–16 | 0 | 100 | 5–10 |
| | | | IgGs | | |
| Healthy donors | 0 | 0 | 0 | 0 | 0 |
| Hepatitis B | 4–5 | 0.02 *(0.3)* | 0.06 | 0 | nd |
| Polyarthritis | 4 | 0.2 *(10)* | 0.02 | 14 (*700*) | 3 (150) |
| HT | 10 | 0.2 *(13*) | 0.016 | 10 *(625)* | 2 (125) |
| SLE | 35 | 0.5–0.8 *(4)* | 0.2 | 4 *(20)* | 5.0 (25) |
| MS | 4–8 | 0.6–4.0 (*8*) | 0.1–0.2 | 5–7 *(43)* | 10–15 (83) |

* All specific activities were estimated per mole of protein. The specific activities of pancreatic RNase A in the hydrolysis of poly(C) and cCMP were taken for 100%; the specific activities of all catalysts in the hydrolysis of poly(U), poly(A), and total yeast RNA was calculated relatively to that for poly(C) hydrolysis by RNase A.

** The ratios of the rates of hydrolysis of the analyzed substrate and poly(A) by the same enzyme or Abz are given in the parentheses.

*** Not determined.

§ Data for three human blood RNases.

substrate specificity similar to that of RNases from bacteria, viruses, and also from cobra and viper venom, which are highly toxic for humans. A change in pH reveals minor Abz subfractions with very different patterns of cleavage of various tRNA; some Abs effectively hydrolyze RNA with comparable rates within the pH range 4.5–9.5. Although the RNase relative activities are strongly dependent on the patient, in general they increase in the following order: hepatitis < polyarthritis < HT < SLE ≤ MS[25,26,27,28,29].

Human milk contains extremely unusual Abzs, which have not been found so far in the sera of AD patients, and their substrate specificities are completely different from those of canonical enzymes. All known protein, lipid, and polysaccharide kinases, including the only milk protein kinase, can use only ATP as the phosphate group donor. Milk sIgA and IgG Abzs phosphorylating about 15 milk proteins as well as unusual minor lipids, oligo- and polysaccharides tightly bound with Abzs can use almost any deoxy- or ribonucleoside-5'-triphosphate, and *ortho*phosphate as a phosphate donor[137,138,139,140,141,142].

In contrast to all canonical nucleases, which have one pronounced pH optimum (6.8–7.5), catalytic SLE IgGs usually have several optima and high activity over a wide pH range (6.0–9.5)[122]. A very significant difference in the number of pH optima distributed between pH 5.0 and 10 or comparable rates at all pHs were also observed for DNase, RNase, and protease Abs from patients with SLE and MS, and MRL-lpr/lpr mice (e.g., Fig. 6)[121,122,136].

It is known that canonical proteases, depending on their biological function, can have one optimal pH ranging from acidic (2.0) to neutral and alkaline (8–10). In contrast to all human proteases, catalytic IgGs demonstrated high hMBP-hydrolyzing activity and several optima within a wide pH range (2.6–10)[126]. A similar situation of several optima over a very wide pH range was recently observed for human milk sIgA hydrolyzing casein, and for integrase-cleaving IgGs and IgMs from HIV-infected patients[152].

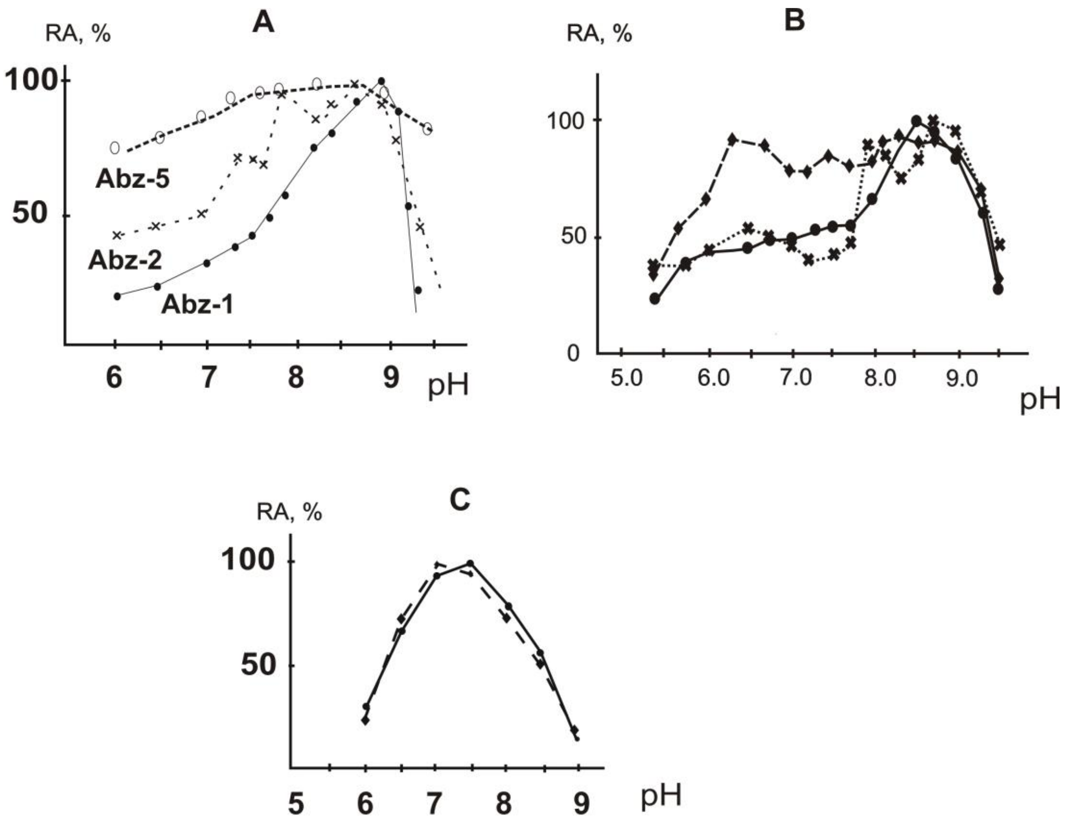

**FIGURE 6**. pH dependence of the relative RNase activity of human blood RNases and IgGs and IgMs from the sera of six SLE patients in the hydrolysis of [5'-$^{32}$P](pA)$_{13}$ oligonucleotide. Results for three electrophoretically and immunologically homogeneous IgGs (A) and IgMs (B), and human sera RNase 3 (•) and RNase 4 (▪) (C), are shown[121,122].

Changing the reaction pH, one can reveal not only the major fraction of Abzs in different individuals, but also other subfractions of Abs, the activities of which at other pHs may be comparable with or less than that of the major subfraction. In addition, the number of $K_m$ and $V_{max}$ values characterizing the interaction of different mABZs and pAbzs with their specific substrates, can significantly increase when they are measured at several pH values.

After Abs purification under standard conditions, the relative amount of metals bound with MS IgGs decrease in the order: Fe ≥ Ca > Cu ≥ Zn ≥ Mg ≥ Mn ≥ Pb ≥ Co ≥ Ni[35], while for milk sIgAs, the order is different: Ca > Mg ≥ Al > Fe ≈ Zn ≥ Ni ≥ Cu ≥ Mn[152]. Abs from individual humans can interact with various metal ions, demonstrating significant differences in the bound metal ions in spite of the comparable content of the main types of metals in the milk and blood plasma. It is known that DNase II is a metal-independent nuclease, while DNase I hydrolyzes DNA only in the presence of $Mg^{2+}$ or some other metal ions[121,122,123]. DNase IgGs and/or IgMs from the sera of patients with SLE, MS, polyarthritis, HT, asthma, hepatitis, HIV and bacterial infections, and immunized rabbits are similar to DNase I in their $Me^{2+}$ dependence; they are activated mainly by $Mg^{2+}$ and $Mn^{2+}$, although some Ab preparations from some individuals are better activated by other metal ions[78,111,121,122,123,143,153]. In contrast to Abzs from autoimmune patients, human milk sIgA is only slightly activated by $Mg^{2+}$, $Mn^{2+}$, or $Zn^{2+}$, and the cleavage of DNA substrates is inhibited by $Ca^{2+}$ and $Cu^{2+}$[119].

The most detailed study of Me-dependent diversity of DNase Abs was carried out for IgGs from MRL-lpr/lpr mice[136]. DNase pIgGs were not active after dialysis against EDTA, but could be activated by several externally added $Me^{2+}$ ions, with the level of activity decreasing in the order: $Mn^{2+} \geq Mg^{2+} > Ca^{2+} \geq Cu^{2+} > Co^{2+} \geq Ni^{2+} \geq Zn^{2+}$, whereas $Fe^{2+}$ did not stimulate hydrolysis of scDNA by the Abs. The data for some metals ions are given in Fig. 7[136]. The dependences of the relative activities on the concentration of

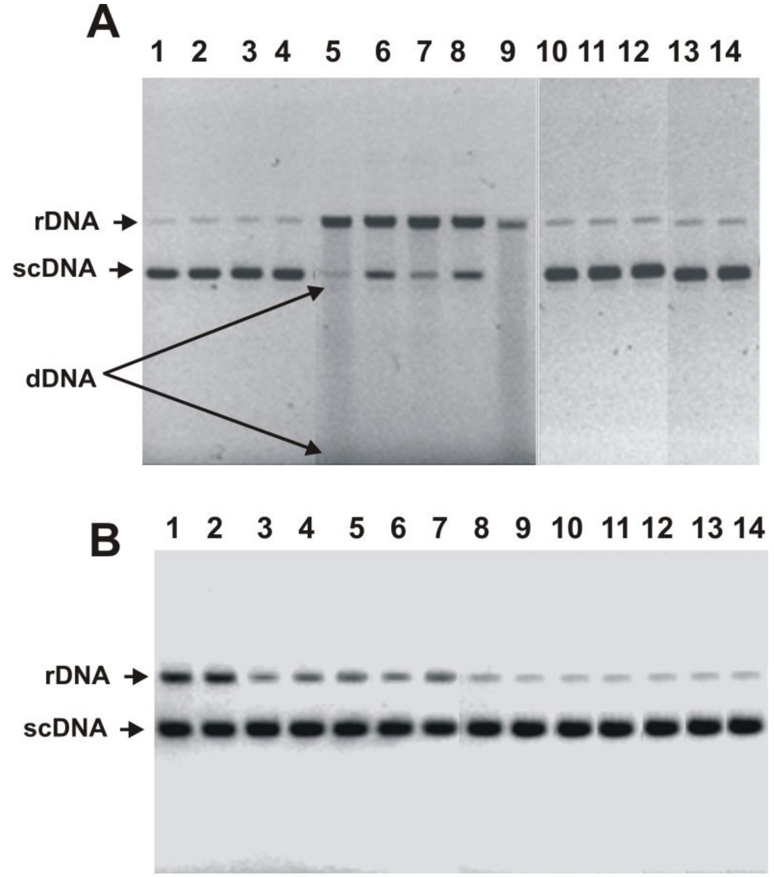

**FIGURE 7**. (A) Cleavage of sc pBluescript DNA after a 30-min incubation in the presence of 0.03 mg/ml IgGs (previously dialyzed against EDTA) from five mice and 2 mM $NiCl_2$ (lanes 5–9) or $FeCl_2$ (lanes 10–14)[136]. Lanes 1 and 2 correspond to scDNA incubated with $NiCl_2$ or $FeCl_2$, respectively, in the absence of Abs; lanes 3 and 4, incubation of scDNA with $NiCl_2$ and $FeCl_2$ in the presence of IgGs from a healthy mouse. (B) IgG-dependent cleavage of scDNA under special conditions leading to a formation of only relaxed DNA (rDNA) in the presence of IgGs from different mice and different $Me^{2+}$ ions (2 mM): 1, $Mg^{2+}$; 2 and 3, $Mn^{2+}$; 4, $Cu^{2+}$; 5, $Ca^{2+}$; 6, $Co^{2+}$; 7, $Ni^{2+}$; 8, $Zn^{2+}$. Lanes 9–14 correspond to the incubation of scDNA without Abs in the presence of different ions (2 mM): 9, $Mg^{2+}$; 10, $Mn^{2+}$; 11, $Cu^{2+}$; 12, $Ca^{2+}$; 13, $Co^{2+}$; 14, $Ni^{2+}$). In order to limit scDNA cleavage only to the plasmid relaxation, the time of incubation was varied from 8 to 30 min, and the Abs were used in the concentrations $2 \times 10^{-5}$ to $1 \times 10^{-3}$ mg/ml.

different $Me^{2+}$ ions are generally bell shaped, demonstrating one to four maxima at different concentrations of $Me^{2+}$ ions in the 0.1–12 mM range, depending on the ion. After separation of DNase Abzs into many fractions on DNA-cellulose, each fraction demonstrates a specific ratio of the relative activities in the presence of different metal ions, and some fractions can be better activated in the presence of combinations of different metal ions[121,122].

A specific Me-dependent fraction hydrolyzing MBP can be obtained by chromatography of MS IgGs on Chelex-100; IgGs do not hydrolyze human MBP in the absence of $Me^{2+}$ ions, but are activated after addition of external $Me^{2+}$ ions: $Mg^{2+} > Mn^{2+} > Cu^{2+} > Ca^{2+}$[35]. IgGs from some MS patients are also activated by other metal ions; $Ni^{2+}$ and, to a lesser extent, $Fe^{2+}$, $Co^{2+}$, $Zn^{2+}$, $Pb^{2+}$, and $Co^{2+}$. Later it was shown that Abs from human milk and from the sera of AIDS patients contain Me-dependent fractions specifically hydrolyzing casein and HIV integrase, respectively[152]. The observed properties of all Abzs distinguish them from other known mammalian metalloproteases and demonstrate their significant catalytic diversity.

Altogether, pAbzs with many catalytic activities from patients with different autoimmune, viral, and bacterial diseases can be Abs of different classes and subclasses (IgG1–IgG4, sIgA1, sIgA2, etc.) containing light chains of λ or κ type. All these Abzs are catalytically very heterogeneous; they can contain subfractions of monoclonal Abzs differing in the structure of catalytic centers (e.g., similar to serine, thiol, acidic, and metalloproteases; Me-dependent and -independent nucleases and proteases; $H_2O_2$-dependent and -independent oxidoreductases), net charge, thermal stability, pH optimum, dependence on different metal ions, substrate specificity, the affinity for substrates, etc. The data strongly suggest that some Abzs may have chimeric active centers, combining parts of the active centers of several different canonical enzymes.

Abs of healthy humans usually do not possess nuclease activities, but a possible number of AGs that can stimulate production of DNase and RNase Abzs in autoimmune patients is significantly greater than those for healthy mammals. First, due to an increased level of apoptosis, Abzs may be produced directly to DNA and its complexes with various proteins. It is known that an immune response to DNA, especially in its complexes with proteins, depends to a certain extent on the DNA sequence and the length of DNA fragments[88,89,90,91,92]. The number of different DNA molecules stimulating the formation of Abs may be very high since the sera of autoimmune mammals contain highly fragmented nuclear DNA from apoptotic cells, and even relatively small DNA fragments of different sequences can trigger immune response. As mentioned above, anti-idiotypic Abs against active centers of different nucleases (and even topoisomerase) can also possess catalytic activity[88,89,90,91,92]. It means that, potentially, any enzyme degrading DNA or RNA can also stimulate formation of DNase Abzs, which can be significantly different in their enzymatic properties. During the spontaneous development of profound ADs, the specific reorganization of the immune system is associated with changes in the differentiation profile of HSCs and with production of different Abzs[101,102,103]. Therefore, depending on the type of autoimmune, viral, or bacterial disease, and specific immune system of every individual, very different Abzs with various structures and biological functions may be produced. Overall, autoimmune mammals can be considered as a promising reservoir of new types of biocatalysts with new and very diverse structural and enzymatic properties.

### ABZYMES IN DIAGNOSTICS AND AS THERAPEUTIC AGENTS

We have shown that the appearance of Abzs specific for various substrates is among the earliest and clearest signs of autoimmune reactions in a number of ADs[25,26,27,28,29]. The catalytic activity of nuclease, ATPase, and MBP-hydrolyzing Abzs usually can be very easily detected at the onset of ADs when the concentrations of Abs to DNA or other auto-AGs are still low and correspond to their ranges in healthy donors. Therefore, assays for some types of Abzs could in principle be used as diagnostic tools for ADs. Since Abzs are usually present in the sera of infected patients, diagnostics cannot rely solely on nuclease Abz analysis. Yet, diseases caused by bacterial and viral infection are diagnosed as a rule by independent specific methods, which should be used to exclude infections if ADs were to be diagnosed by Abz assays. The major fractions of RNase Abzs from the sera of patients with different ADs demonstrate different and quite specific patterns of cleavage of $tRNA^{Phe}$ and some other tRNAs[129,130,131,132,133]. This criterion can add to more precise diagnostics of autoimmune pathology (SLE, MS, polyarthritis, HT, viral hepatitis, etc.). In addition, a comparison of the substrate specificity of RNase Abzs toward poly(A), poly(U), poly(C), and poly(G) can also distinguish different types of autoimmune pathologies[130,131]. MBP-hydrolyzing Abzs and Abs hydrolyzing viral reverse transcriptase and integrase can be used in diagnostics of MS and AIDS, respectively[33,34,35,36,37]. In addition, Abzs offer good indicators of alterations of autoimmune processes in different ADs.

Monoclonal Abs are suitable for therapeutic applications due to their excellent target-binding characteristics (specificity, affinity) and long half-life *in vivo*. Abzs can be developed into a new generation of therapeutics with enhanced AG inactivation capability. The field of monoclonal Abzs with immunotherapeutic potential has recently been reviewed[154,155,156,157,158,159,160,161]. Abs and Abzs can be used to neutralize pathogens, toxins, and endogenous mediators of pathology, and employed for new methods of drug synthesis, as well as for *in vivo* therapies[157]. For instance, cocaine-hydrolyzing Abzs have been developed and may provide a novel approach to the problems of drug addiction[158,162]. Abzs that cleave HIV envelope gp120 protein may find their use in the treatment of AIDS patients[155,158,160]. The properties of the Abzs can provide valuable guidance in designing a prophylactic vaccine that amplifies the protective catalytic immunity to HIV[155,158,160]. Abzs specifically hydrolyzing viral reverse transcriptase and integrase[36,37] are potentially interesting for designing new anti-HIV agents.

The appearance of factor VIII (FVIII)–neutralizing Abs in hemophilic patients following therapeutic administration of exogenous FVIII is one of the major factors complicating the treatment of this disease[163,164,165]. However, recent data suggest that some FVIII inhibitors may be catalytic Abs hydrolyzing FVIII. The characterization of these Abs as site-specific proteases may provide new approaches to the treatment of hemophilia[165,166,167]. Abs that bind amyloid β peptide (Aβ) are under clinical trials for immunotherapy of Alzheimer's disease[154,168]. Hydrolysis of peripheral Aβ by the IgMs may induce increased Aβ release from the brain. The catalytic IgMs are increased in patients with Alzheimer's disease, presumably reflecting a protective autoimmune response. Reduced Aβ aggregation and neurotoxicity attributable to the catalytic function are evident. These findings provide a foundation for a development of catalytic Igs for Alzheimer's disease immunotherapy[168]. Monoclonal Abzs catalyzing specific degradation of the β subunit of *Helicobacter pylori* urease have been developed[165]. The heavy chain of Abzs also exhibits the specific degradation of urease. These Abzs were reported to be prepared for medicinal use. Abzs with oxidoreductase activities[112,113,114,115,116] can be used for detoxification of reactive oxygen species and oxidation of different mutagenic and carcinogenic compounds in human blood.

In conclusion, a number of studies of Abzs show the extremely wide potential of the immune system in producing Abzs possessing very different enzymatic activities, which very often are not comparable with those of known enzymes, and natural Abs with specified and novel functions may have wide potential for biotechnology and medicine.

### ACKNOWLEDGMENTS

This research was made possible in part by grants from the Presidium of the Russian Academy of Sciences (Molecular and Cellular Biology Program), the Russian Foundation for Basic Research, and the Siberian division of the Russian Academy of Sciences.
